# Complementary and Alternative Medicine for Diabetes 2014

**DOI:** 10.1155/2015/685248

**Published:** 2015-03-26

**Authors:** Wen-Chin Yang, Cicero L. T. Chang, Cheng-Rui Li, Srinivas Nammi, William C. S. Cho

**Affiliations:** ^1^Agricultural Biotechnology Research Center, Academia Sinica, Taipei 11501, Taiwan; ^2^Department of Veterinary Medicine, National Chung Hsing University, Taiwan; ^3^Sanford-Burnham Medical Research Institute, La Jolla, CA 92037, USA; ^4^School of Science and Health, University of Western Sydney, Sydney, NSW 2751, Australia; ^5^NICM Centre for Complementary Medicine Research, University of Western Sydney, Sydney, NSW 2751, Australia; ^6^Department of Clinical Oncology, Queen Elizabeth Hospital, Hong Kong

Diabetes, one of the incurable pandemic diseases, is characterized by insulin deficiency and insulin resistance, leading to aberrant homeostasis of glucose, protein, and lipid. Genetic and environmental factors are the primary causes of diabetes. International Diabetes Federation estimated that 400 million people are afflicted with this disease worldwide. However, current oral antidiabetic agents commonly used in orthodox medicine have unmet efficacy and undesirable side effects in patients, which, consequently, can develop cardiovascular diseases, retinopathy, neuropathy, nephropathy, foot ulcers, and so forth. Therefore, there is an urgent need for development of new remedies for diabetes.

World Health Organization estimates that 80% of the world population uses complementary and alternative medicine (CAM) for their primary health care. Therefore, CAM, including medicinal herbs, acupuncture, moxibustion, and other therapies, is an extraordinary source of diabetes therapy. The goal of this special issue was to compile and update the advancement made on basic and clinical research into CAM for diabetes and its complications.

This special issue accepted 8 manuscripts out of 36 contributions. They cover a wide range of topics, from preclinical studies to clinical trials on CAM for diabetes and its complications. One study by K. Raafat and W. Samy titled “Amelioration of Diabetes and Painful Diabetic Neuropathy by* Punica granatum* L. Extract and Its Spray Dried Biopolymeric Dispersions” describes the effect of ethanol extract (Pg), casein/Pg dispersion (F1), chitosan/Pg dispersion (F2), and gallic acid (GA) on diabetes and related neuropathy in mice treated with alloxan. They first established the diabetic mice whose *β* cells were destroyed by alloxan. As expected, those mice had hyperglycemia. In contrast, Pg, F1, F2, and GA significantly lowered blood glucose in the mice. Besides, they also tested the impact of the extracts and compound on neuropathy. The data showed that Pg, F1, F2, and GA improved the diabetic neuropathy as evidenced by tail flick and hot plate latency tests. In the paper entitled “Effects of Dietary Supplementation with* Agaricus sylvaticus* Schaeffer on Glycemia and Cholesterol after Streptozotocin-Induced Diabetes in Rats,” M. B. Mascaro et al. report the protective action of the mushroom,* A. sylvaticus*, as shown by plasma biochemical parameters and islet structure in streptozotocin-treated rats. Animal studies indicated that the* A. sylvaticus* crude extract reduced the levels of cholesterol, HDL, triglycerides, blood sugar, GPT, and alkaline phosphatase but increased those of transferrin and urea in streptozotocin-treated rats. Histochemical staining data showed that* A. sylvaticus* suppressed islet atrophy. The overall data imply that* A. sylvaticus* protected against type 1 diabetes probably via hepatic and pancreatic regulation. In the paper “Immunomodulatory and Antidiabetic Effects of a New Herbal Preparation (HemoHIM) on Streptozotocin-Induced Diabetic Mice,” J.-J. Kim et al. studied the beneficial effect of the herbal preparation (HemoHIM), composed of* Angelica gigas* Nakai,* Cnidium officinale* Makino, and* Paeonia japonica* Miyabe, on type 1 diabetes in streptozotocin-treated mice. They showed that HemoHIM treatment reduced hyperglycemia, improved glucose tolerance, and increased blood insulin. Besides, this treatment could restore the thymus weight, the number of blood white cells, blood lymphocytes, splenic CD4 and CD8 T cells, and ConA-stimulated IFN*γ* production. Taken together, the data suggest that HemoHIM exerts antidiabetic action via regulation of blood glucose homeostasis and immunity. Another study by Y.-H. Kuo et al., “Caffeamide 36-13 Regulates the Antidiabetic and Hypolipidemic Signs of High-Fat-Fed Mice on Glucose Transporter 4, AMPK Phosphorylation, and Regulated Hepatic Glucose Production,” reports that caffeamide 36-13 (TS), a caffeic acid amide, improved hyperglycemia and hyperlipidemia in high fat diet-fed mice as evidenced by the level of blood glucose, triglyceride, insulin, leptin, and free fatty acid (FFA). Accordingly, TS diminished abdominal visceral fat, cell size of adipocytes in visceral fat, and hepatic triglyceride contents. Moreover, TS increased the level of glucose transporter 4 (GLUT4) in skeletal muscle, Akt phosphorylation, and AMPK phosphorylation in liver and/or muscle. In contrast, this treatment reduced the expression level of phosphoenolpyruvate carboxykinase (PEPCK), glucose-6-phosphatase (G6Pase), fatty acid synthase (FAS), and apolipoprotein C-III (apo C-III) in liver. Overall, this work postulates that TS possesses glucose- and lipid-lowering activities via a reduction of hepatic glucose reduction involving reduced PEPCK and G6Pase and a reduction of FAS and apo C-III, respectively. In the paper entitled “Protein Fractions from Korean Mistletoe (*Viscum album coloratum*) Extract Induce Insulin Secretion from Pancreatic Beta Cells,” K.-W. Kim et al. show that* V. album coloratum* extract (KME) stimulated insulin secretion in RINm5F, a *β* cell line. Furthermore, DEAE fraction of KME extract, obtained from DEAE chromatography, increased the mRNA level of insulin and PDX1. Both protein fractions, lectin-free fraction and DEAE fraction of KME, could reduce blood glucose in alloxan-treated mice. This study implies that KME can exert antidiabetic action via upregulation of insulin production.

In human clinical studies, Lai and his colleagues (“*Bidens pilosa* Formulation Improves Blood Glucose Homeostasis and Beta-Cell Function in Men: A Pilot Study,”) reports a pilot study of the antidiabetic herb,* B. pilosa*, in diabetic patients. The authors first showed that single or combination use of* B. pilosa* could significantly reduce fasting blood glucose and glycosylated hemoglobin A1c (HbA1c) in diabetic subjects. Consistently,* B. pilosa* increased fasting insulin. Moreover, combination use of* B. pilosa* with other antidiabetic agents had better glucose control than* B. pilosa* alone. The homeostatic model assessment (HOMA) data proposed that the antidiabetic activity of* B. pilosa* was via preservation of *β*-cell function. They also showed safety of the* B. pilosa* formulation in healthy subjects.

In addition to original research articles, this special issue also features review articles. A review entitled “Naturally Occurring Anthraquinones: Chemistry and Therapeutic Potential in Autoimmune Diabetes” describes a class of naturally occurring anthraquinones (NOAQs) in relation to type 1 diabetes from chemical, biological, pharmacological, and clinical aspects. Another review by R. D. Umrani and K. M. Paknikar, “Jasada Bhasma, a Zinc-Based Ayurvedic Preparation: Contemporary Evidence of Antidiabetic Activity Inspires Development of a Nanomedicine,” summarizes the importance of one Ayurvedic medicine, zinc-based bhasma, on diabetes. The authors discuss the preparations of zinc-containing bhasma in a nanoparticle size and their studies in cells and animals. The zinc-containing bhasma might act to delay the severity of diabetes and progression of its complications. These review articles shed light on further directions for CAM in diabetes and diabetic complications.

We anticipate that this special issue will be of interest to the researchers/readers in the field of diabetes and draw more attention to the use of CAM-based therapies for diabetes and the related complications.

